# Risk factors for orthodontic mini-implants in skeletal anchorage biological stability: a systematic literature review and meta-analysis

**DOI:** 10.1038/s41598-020-62838-7

**Published:** 2020-04-03

**Authors:** María Dolores Casaña-Ruiz, Carlos Bellot-Arcís, Vanessa Paredes-Gallardo, Verónica García-Sanz, José Manuel Almerich-Silla, José María Montiel-Company

**Affiliations:** Stomatology Department, University of Valencia, Valencia, Spain

**Keywords:** Health care, Dental treatments, Dental treatment planning

## Abstract

The reason of the biological stability loss of mini-implants is still a matter of discussion between dentistry professionals. The main objective of this systematic literature review and meta-analysis was to analyze the risk factors that prejudice this loss. A search was made in the electronic databases Pubmed, Scopus, Embase and Cochrane, in addition a manual search was made too in Grey Literature (Opengrey). No limits were set on the year of publication or language. The inclusion criteria were: studies in humans treated with fixed appliances with mini-implants, where the risk factors for secondary stability were evaluated for a minimum of 8 weeks. After eliminating duplicate studies and assessing which ones achieve the inclusion criteria, a total of 26 studies were selected for the qualitative synthesis, 18 of them were included in the quantitative synthesis. Common risk variables were compared in all of them. Analyzing the forest and funnel plots, statistically significant differences were obtained only for location, the upper maxilla having lower risk than the mandible with an odds ratio of 0.56 and confidence interval of 0.39 to 0.80. Prospective studies under controlled conditions should be required in order to obtain a correct assessment of the variables analyzed.

## Introduction

The use of mini-implants to provide anchorage for force application has simplified and improved the efficacy of many orthodontic treatments, reducing unwanted movements of other teeth, especially in adult patients. For this reason, anchorage in bone is becoming a much more widely used technique^1^.

Mini-implants are made of titanium or stainless steel, their structure has three different parts: the top one (supra-gingival, which allows the anchor), the medium area or neck, and the thread, which is inserted inside the bone and provides mechanic anchor. Moreover, the most common types of mini-implants are: Mini Implant System (dbOrthodontics), Vector Temporary Anchorage System (Omco) and the Abso Anchor System (Dentos). All of them are available in different lengths (6–12 mm) and diameters (1–2 mm)^2^.

Mini-implants or TADs (Temporary Anchorage Devices)^2^ have been used mainly as an auxiliary fixing method but more recently have been adopted for additional functions such as: mid-line or inclined plane alignment, space opening, or molar intrusion or extrusion^3^. The increasing use of mini-implants is justified by its multiple advantages, the simplicity of the surgical technique, and low cost. Moreover, it is well accepted by patients and achieves success rates of 80–90%^4^. While failure may be avoided by establishing the right therapeutic protocols, the fact that a percentage of mini-implants fail should not be overlooked.

Stability refers to the resistance to reactive forces, offered by teeth or other oral or extraoral structures, that would lead to unwanted movements^5^. In the case of mini-implants, two types of stability can be distinguished: primary and secondary. Primary stability is mechanical and is achieved by the mini-implant compressing the bone during insertion, while secondary, or biological stability, begins at the moment of placement and increases during the bone remodeling or healing process^6^.

The transition from primary to secondary stability takes at least 8 weeks. In this period of time, the failure of mini-implants can occur. A mini-implant failure is regarded as the appearance of mobility (eventual loss), inflammation or infection. Although biological stability is reached by orthodontic mini-implants when these are successful, osseointegration does not occur in this type of implants since they are temporary devices^6^.

Some controversy exists as to which risk factors affect mini-implant secondary stability^7^. The aim of this systematic literature review and meta-analysis was to analyze the variables affecting biological stability of mini-implants use for orthodontic anchorage.

The primary hypothesis of the present meta-analysis was that multiple factors can affect secondary or biological stability: patient-dependent (age, sex, hygiene), mini-implants-dependent (length and diameter) or technique-dependent (clinical experience, location, time, predrilling or not). The null hypothesis was that there are not any variables that significantly affect biological stability of mini-implants.

## Materials and Methods

### Review question

Meta-analysis was performed following PRISMA (Preferred Reporting Items for Systematic Reviews and Meta-Analyses) guidelines^8^. The review protocol was registered in the PROSPERO database (reg. no. CRD42018083900). The review question of the present study was the following: in patients treated with fixed appliances (brackets) and mini-implants, what are the risk factors for secondary stability?

PICO(S) elements^9^ included in our review can be defined as follows: Patients treated with orthodontic fixed appliances (P) where mini-implants were placed as auxiliary devices (I) in different locations (anterior/posterior upper maxilla, anterior/posterior mandible), different ages and genders and different lengths and diameters of the mini-implants (C) and where the secondary stability of this temporary devices had been analyzed (O). Randomized clinical trials, longitudinal studies, cohort and case/control studies were eligible to be included (S).

### Eligibility criteria

“Articles” and “Articles in press” were included: randomized clinical trials (RCT), longitudinal studies, cohort or case/control studies both retrospective and prospective. No restrictions were applied regarding the year of publication or language. Inclusion criteria were: human studies that evaluated risk factors for secondary stability of mini-implants used in orthodontic treatment for a period of at least 8 weeks.

### Outcome

Secondary, or biological stability, begins at the moment of placement and increases during the bone remodeling or healing process. Biological stability is lost when mobility, severe inflammation or infection appear.

### Information sources, search strategy, and study selection

To identify relevant articles, an electronic search was conducted in four databases: Pubmed, Embase, Scopus and Cochrane. A manual search was conducted in Opengrey literature. In some cases, the authors were contacted by e-mail to request additional information. The key search terms used to identify articles were: (Humans/human OR patients OR adults OR male OR female) AND (mini-screws OR micro-screws OR mini-implants OR temporary anchorage devices OR TAD* OR stability OR long-term stability OR skeletal-anchorage OR orthodontic treatment) AND (orthodontic failure-rates OR orthodontic success-rates). Two reviewers (MDC-R and CB-A), assessed the titles and abstracts of the articles identified in the electronic search; whenever disagreement occurred a third reviewer was consulted (JMM-C). If the abstract did not provide sufficient information to reach a decision to include it in analysis, the reviewers read the full text. Then, the full texts of the articles selected were read, and if they failed to meet inclusion criteria, the reasons for rejection were recorded. (Table 1).

**Table 1:** Records excluded and reasons for exclusion.

RECORDS EXCLUDED	ARTICLES EXCLUDED, WITH REASONS
Männchen *et al*., 2008	*No answer* research question.
Ji *et al*., 2008	*No answer* research question.
Tsoudis *et al*., 2008	*Study design not considered expedient (literary review)*.
Reynders *et al*., 2009	*Study design not considered expedient (literary review)*.
Antoszewska *et al*., 2009	*No answer* research question.
Schätzle *et al*., 2009	*Study design not considered expedient (literary review)*.
Schätzle *et al*., 2010	*Study design not considered expedient (literary review)*.
Kim *et al*., 2010	*No answer* research question.
Papadopoulus *et al*., 2011	*Study design not considered expedient (literary review)*.
Min *et al*., 2012	*No answer* research question.
Consolaro *et al*., 2014	*Study design not considered expedient (literary review)*.
Kuroda *et al*., 2014	*Study design not considered expedient (literary review)*.
Rodriguez *et al*., 2014	*Study design not considered expedient (meta-analysis)*.
Dalesandri *et al*., 2014	*Study design not considered expedient (meta-analysis)*.
Romano *et al*., 2015	*Study design not considered expedient (literary review)*.
Uribe *et al*., 2015	*Study design not considered expedient (literary review)*.
Cornelis *et al*., 2015	*Study design not considered expedient (book chapter)*.
Papadopoulus *et al*., 2015	*Study design not considered expedient (book chapter)*.
Sarul *et al*., 2015	*Study design not considered expedient (literary review)*.
Lin *et al*., 2015	*Study design not considered expedient (literary review)*.
Liu *et al*., 2016	*Study design not considered expedient (literary review)*.
Kim *et al*., 2016	*Study design not considered expedient (literary review)*.
Leo *et al*., 2016	*Study design not considered expedient (literary review)*.
Hong *et al*., 2016	*Study design not considered expedient (literary review)*.
Yi *et al*., 2016	*Study design not considered expedient (literary review)*.
Afrashtehfer *et al*., 2016	*Study design not considered expedient (meta-analysis)*.
Yi *et al*., 2017	*Study design not considered expedient (meta-analysis)*.
Alharbi *et al*., 2018	*Study design not considered expedient (meta-analysis)*.
Mohammed *et al*., 2018	*Study design not considered expedient (meta-analysis)*.
Lyczek *et al*., 2019	*Study design not considered expedient (literary review)*.
Azeem *et al*., 2019	*Primary stability*
Azeem *et al*., 2019	*Primary stability*
Kakali *et al*., 2019	*Study design not considered expedient (literary review)*.
Ichsnoke *et al*., 2019	*No answer* research question.

### Data items and collection

The review and meta-analysis was updated for the last time on January 4^th^ 2020. The following variables were extracted from the works selected: author; year of publication; study type; sample size; demographic variables (patient age and sex); type of mini-implant (length and diameter); mini-implant location (maxillary or mandibular); position (left or right, vestibular, lingual/palatine, or crestal); and the number of mini-implants per patient. (Table 2)

**Table 2:** Study data. Analyzed variables: study design, diameter, length, patient, age, total number of TADs, position, number of success TADs, definition of success and definition of failure.

AUTHOR YEAR	STUDY DESIGN	DIAMETER (MM)	LENGTH (MM)	PATIENT (N)	AGE	N° of TAD	POSITION	N° of SUCCESS	DEFINITION OF SUCCESS	DEFINITION OF FAILURE
Miyawaki *et al*., 2003	Retrospective	1.0 /1.5/2.3	6 / 11/ 14	51	21.8+_ 7.8	134	Upper jaw. 63 Lower jaw. 61	103	NA	Diameter 1 mm or less, inflammation periimplant tissue, high mandibular plane.
Chen *et al*., 2004	Prospective	2.0	5/7/9/11/13/15	44	29 _+ 8.9	140	Upper jaw. 105 Lower jaw. 35	125	Absence of inflammation and clinical movement.	NA
Park *et al*., 2006	Retrospective	1.2/ 1.2/ 1.2/ 2	5/ 6,8,10/ 4,6,8,10/ 10,12,14,15	87	15.5 _+ 8.3	227	Upper jaw. 124 Lower jaw. 103	208	Keep until the final of the treatment. Try to remove.	Lost during the treatment.
Chen *et al*., 2007	Retrospective	2/ 2/ 1.2	5–9/ 5–21/ 4–10	129	24.5	359	Upper jaw x. 263 Lower jaw. 96	306	Enough stability during the treatment.	Lost during the treatment.
Wiechmann *et al*., 2007	Prospective	1.1 /1.6	5/ 6/ 7/ 8/ 10	49	26.9 _+ 8.9	133	Upper jaw. 88 Lower jaw. 45	102	Absence of inflammation and clinical movement.	NA
Kuroda *et al*., 2007	Retrospective	1.3/ 2.0/ 2.3	6 /7/ 8/ 10/ 11/ 12	75	21.8 _+ 8.2	79	Upper jaw. 156 Lower jaw. 60	70	Steady during one year.	NA
Chen *et al*., 2008	Retrospective	2.0	5–9/ 8–14	194	25.1	489	Upper jaw. 399 Lower jaw. 90	445	NA	Lost during the treatment.
Moon *et al*., 2008	Retrospective	1.6	8	209	20.3	480	Upper jaw. 279 Lower jaw 0.201	402	Absence of mobility after 8 month.	NA
Wu *et al*., 2009	Retrospective	1.1–1.5/ 1.7/ 2.0	7/ 8/ 10/ 11/ 12/ 13/ 14/ 15	166	26.5_+ 8.9	414	Upper jaw. 268 Lower jaw. 135	372	NA	Lost after 8 month, or fractured after the insertion.
Viwattanatipa *et al*., 2009	Prospective	1.2	8/ 10/ 12	49	23.2	97	Upper jaw. 97 Lower jaw. 0	65	NA	Mobility, displacement or infection.
Motoyoshi *et al*., 2009	Retrospective	1.6	8	52	26.1_+ 8.4	109	Upper jaw. 42 Lower jaw. 67	103	No mobility, no lost during the treatment.	NA
Lee *et al*., 2010	Prospective	1.8	8.5	141	27	260	Upper jaw. 260 Lower jaw. 0	238	NA	NA
Moon *et al*., 2010	Retrospective	1.6	8	306	14.45_+ 2.64 23.73_+ 2.70 37.04_+ 7.26	778	NA	614	Steady during one year.	NA
Manni *et al*., 2010	Retrospective	1.5/ 1.3	9/ 11	132	25.9	300	Upper jaw. 427 Lower jaw. 351	243	Absence of inflammation or loss.	Inflammation and instability.
Takaki *et al*., 2010	Retrospective	NA	NA	455	25.7 _+ 9.8	904	Upper jaw. 265 Lower jaw. 639	842	NA	Mobility or loss of the implant.
Sharma *et al*., 2011	Retrospective	1.3	8	73	22.45	139	Upper jaw. 97 Lower jaw. 42	122	Absence of inflammation and clinical movement. Keep until the final of the treatment	Spontaneous loss, severe mobility, replacement, infection, pain, pathology of soft tissue.
Topouzelis *et al*., 2012	Retrospective	1.2/ 1.4	8/ 10	34	27.2_+ 7.3	82	Upper jaw. 62 Lower jaw. 20	74	Absence of inflammation, pain or mobility.	Infección or mobility. Instability with orthodontics forces.
Kim *et al*., 2012	Retrospective	1.6	6/ 8	286	10–30	429	Upper jaw. 357 Lower jaw. 72	332	NA	Mobility or loss after 6 months.
Dobranski *et al*., 2014	Prospective	1.6/ 1.8	6/8/10	166	25.8	293	Upper jaw. 259 Lower jaw. 34	256	NA	Loss of stability.
Yao *et al*. 2015	Retrospective	NA	NA	220	29.3	727	Upper jaw. 412 Lower jaw.231	643	NA	Mobility or loss during the treatment.
Melo *et al*., 2016	Retrospective	1.3/ 1.4/ 1.6	5/ 7/ 9/ 11	570	42.7	1356	Upper jaw. 816 Lower jaw. 539	1208	NA	Clinical mobility or o fracture during the insertion.
Jing *et al*., 2016	Retrospective	1.4/ 2.0	6/ 8/ 10	114	19.26_+ 9.19	253	Upper jaw. 170 Lower jaw. 83	224	Keep until the success.	NA
Lee *et al*., 2016	Retrospective	1.2/ 1.3	8	71	19.2_+ 6.63	127	NA	108	Keep the insertion in the bone with success during one year.	NA
Tsai *et al*., 2016	Prospective	1.5/2	8/9/10/11/12	139	25,7 + − 7,5	254	Upper jaw. 213 Lower jaw. 41	218	NA	NA
Aly *et al*. 2018	Prospective	1.5/1.6/1.8	6/8710	82	21.41	180	Upper jaw. 52 Lower jaw. 128	148	Being functionally stable until the end of the treatment with no signs of inflammation or any pathological condition around the TAD site, and anchorage function sustained until the end of the purpose	Sudden spontaneous loss or the presence of mobility or looseness during routine visits that required replacing the TAD used, or infected painful pathological condition that could be seen as normal inflammation.
Park *et al*., 2018	Retrospective	1.2/ 1.3	8	80	17.95_+ 6.13	160	NA	136	Keep the insertion in the bone with success during one year.	

MM: millimeters; N: number of total patients; TAD: temporary anchorage device; NA: not applicable.

### Risk of bias/quality assessment in individual studies

The quality of the studies was assessed by the same reviewers independently, using the Newcastle-Ottawa scale^10^. In case of any discrepancies in quality assessment, consensus was reached between the reviewers and if this was not possible a third reviewer was consulted. **(**Table 3**)**

**Table 3:** Quality assessment. Newcastle-Ottawa scale^10^.

Author. Year	Selection				Comparability		Outcome			Total
	1	2	3	4	5a	5b	6	7	8	
Miyawaki *et al*., 2003	*	NA	*	*	*		*	*	*	7/9
Chen *et al*., 2004	*	NA	*	*	*		*	*	*	7/9
Park *et al*., 2006	*	NA	*	*	*		*	*	*	7/9
Chen *et al*., 2007	*	NA	*	*	*		*	*	*	7/9
Wiechmann *et al*., 2007	*	NA	*	*	*		*	*	*	7/9
Kuroda *et al*., 2007	*	NA	*	*	*		*	*	*	7/9
Chen *et al*., 2008	*	NA	*	*	*		*	*	*	7/9
Moon *et al*., 2008	*	NA	*	*	*		*	*	*	7/9
Wu *et al*., 2009	*	NA	*	*			*	*	*	6/9
Motoyoshi *et al*., 2009	*	NA	*	*	*		*	*	*	7/9
Viwattanatipa *et al*., 2009	*	NA	*	*	*		*	*	*	7/9
Lee *et al*., 2010	*	NA	*	*	*		*	*	*	7/9
Takaki *et al*., 2010	*	NA	*	*			*	*	*	6/9
Moon *et al*., 2010	*	NA	*	*	*		*	*	*	7/9
Manni *et al*., 2010	*	NA	*	*	*		*	*	*	7/9
Sharma *et al*., 2011	*	NA	*	*			*	*	*	6/9
Topouzelis *et al*., 2012	*	NA	*	*	*		*	*	*	7/9
Kim *et al*., 2012	*	NA	*	*	*		*	*	*	7/9
Dobranski *et al*., 2014	*	NA	*	*	*		*	*	*	7/9
Yao *et al*., 2015	*	NA	*	*	*		*	*	*	7/9
Melo *et al*., 2016	*	NA	*	*	*		*	*	*	7/9
Jing *et al*., 2016	*	NA	*	*	*		*	*	*	7/9
Lee *et al*., 2016	*	NA	*	*	*		*	*	*	7/9
Tsai *et al*., 2016	*	NA	*	*	*		*	*	*	7/9
Aly *et al*. 2018	*	NA	*	*	*		*	*	*	7/9
Park *et al*., 2018	*	NA	*	*	*		*	*	*	7/9

### Summary measures and approach to synthesis

Means and confidence intervals were calculated for patient age, mini-implant length and diameter. Adverse events (loss of mini-implant stability) were recorded in relation to possible risk factors: mandibular or maxillary placement; vestibular, palatine/lingual, or crestal placement; placement on the left or right sides, in anterior or posterior regions; patient age and sex; mini-implant length and diameter.

### Statistical analysis

For the quantitative synthesis odds ratios and 95% confidence intervals of risk factors were calculated, together with their confidence intervals. Heterogeneity was assessed by the Q test and the I2 statistic. A Q test p-value <0.1 was considered heterogeneous. The random effects method was used to calculate the odds ratios. Publication bias was analyzed using funnel plots and Egger’s regression intercept method. The software employed was comprehensive meta-analysis V 3.0 Biostat.

## Results

### Study selection and characteristics

**Figure 1:**
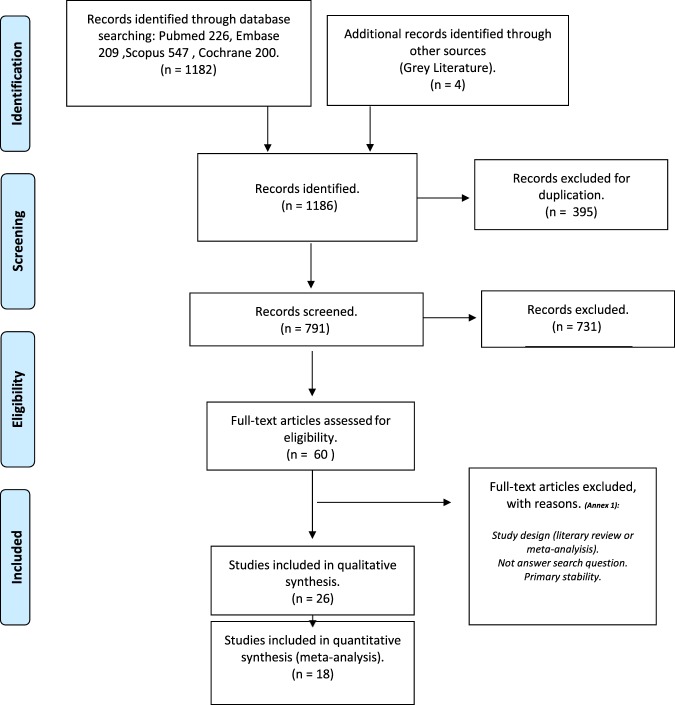
The PRISMA flow diagram^8^.

The electronic search in four databases identified 1182 articles: 226 in Pubmed, 547 in Scopus, 209 in Embase, and 200 in Cochrane; the grey literature manual search located four further works. Duplicates were eliminated leaving a total of 791 studies for analysis. After reading the titles and abstracts of the selected papers, 731 articles were discarded leaving 60 studies for thorough assessment. Full texts of these 60 studies were revised and 34 articles were eliminated as these were literature reviews or meta-analyses or because they failed to answer the research question or did not meet inclusion criteria **(**Table 1**)**. A total of 26 articles fulfilled all inclusion criteria (qualitative synthesis), 18 of which were used for quantitative synthesis (meta-analysis). The PRISMA flow diagram **(**Fig. 1. ***Study selection and characteristics)*** illustrates the entire selection process.

### Results of individual studies, meta-analysis and additional analyses

Patient sample sizes in the articles selected ranged from a minimum of 34^11^ to a maximum of 570^12^, although most had sample sizes averaging around 150 patients. As for distribution by sex, there were more women than men. For example, one study^13^ included 295 women and 64 men, while another study^14^ included 39 women and 5 men. In most of the studies mean patient age was around 25 years, samples generally consisting of predominantly young adults. Of the 26 articles, 7 were prospective studies^15–21^ and 19 retrospective^11–14,22–36^. Follow-up periods ranged from approximately 18 months to 2 years. The shortest follow-up period was 4 months^30^ and the longest 10 years^26^.

Analyzing quality with the Newcastle-Ottawa scale, scores of 6/9 or 7/9 were obtained, which indicated moderate or moderate-high quality respectively. **(**Table 2**)**

### Qualitative synthesis

With regard to the number of mini-implants, high variability was found, one of the study using a total of 1,356 mini-implants^12^ (study with the greatest number of mini-implants within this review) contrarily to another study that used 79 mini-implants, being the study with the lowest number of mini-implants^28^. Most works used an average of around 350 mini-implants. The analysis of mini-implant location found more common placement in the maxilla than in the mandible, excepting 3 studies^21,25,26^. Length and diameter were also evaluated, finding that the most widely used mini-implants were of 8 mm in length and 1.4 mm diameter. Mini-implants of different diameters were used ranging from 1.0 mm^27^ to a maximum of 2.3 mm^27^. As for length, the minimum length used was 5 mm^12^ and the maximum 21 mm^13^. Among studies that provided information about the side on which mini-implants were placed (left or right), only one study placed the same number of mini-implants on both sides^18^; in the other studies, the right side received higher number of mini-implants^17,22,31,34,35^. When mini-implant positions (vestibular, lingual/palatine, or crestal) were compared, the most usual position for mini-implant placement was the vestibular area^33^, with the exception of one article where a higher number of mini-implants were placed in lingual areas^11^.

### Quantitative synthesis

The failure rate calculated from the total of mini-implants placed was analyzed for the risk factors studied. The highest values were observed for ages under 30 years with 19.7%, the mandibular location 18.8% and length higher than 8 mm 18.6% (Table 4).

**Table 4:** Risk factors and event rate.

Risk Factor	EVENT RATE (%)	IC 95%LOWER LIMIT	UPPER LIMIT	Q-VALUE	P-VALUE	I2 (%)	N TOTAL	NUMBER OF STUDIES INCLUDIED
<30 yr-old	19.7	12.9	28.8	219.5	<0.001	96.4	2934	8
Lower jaw	18.8	14.2	24.5	105,7	<0.001	87.7	2433	14
Length > 8 mm	18.6	8.4	36.4	36.2	<0.001	88.9	729	5
Right side	17.3	12.7	23.0	23.2	0.001	74.1	930	7
Anterior	15.7	9.4	25.1	24.6	<0.001	87.8	653	3
Women	15.5	12.6	18.9	123.9	<0.001	87.1	4541	16
Diameter > 1.4 mm	15.3	10.1	22.5	26.1	<0.001	84.7	1591	5
Ridge	15.1	10	22.3	6.655	0.248	24.87	258	6
Men	15.0	12.0	18.5	63.9	<0.001	74.9	1715	16
Length < 8 mm	13.6	9.6	18.9	13.5	0.009	70.3	1239	5
Left side	13.4	9.5	18.7	22.4	0.001	73.2	920	7
Palate	13.4	9.8	18	13.2	0.068	46.8	600	7
Vestibular	12.5	10.3	15.0	24.1	0.001	70.9	2544	7
Posterior	12.1	7.8	18.3	6.7	0.080	55.6	618	3
Upper jaw	11.4	9.1	14.4	65.4	0.000	80.1	3550	14
Diameter < 1.4 mm	10.8	8.9	12.9	0.111	0.991	0.000	912	4
>30 yr-old	9.5	7.4	12.3	10.4	0.234	23.5	755	8

Mini-implant location in the maxilla or mandible showed a significant relation with secondary stability; the odds ratio obtained in the forest plot of this meta-analysis was 0.56 (95% confidence interval (CI) between 0.39 and 0.80), which indicates that mandibular mini-implant placement is a protective factor. Moreover, the I2 value (I2 = 78%) indicated heterogeneity (Q = 63.72; p < 0.001). Analysis of the age variable found that ages of 30 or lower were also a protective factor for mini-implant secondary stability although this did not reach statistical significance, with an odds ratio of 1.59 and 95% CI of 1.01 to 2.50. The I2 value in this case was 63.67%, indicating moderate heterogeneity (Q = 22.02; p = 0.005).

Gender did not show a statistically significant relation with secondary stability, obtaining an odds ratio of 1.40 and 95% CI of 0.96 to 2.04; the I2 value indicated heterogeneity 79.4 (Q = 77.8; p < 0.001). The same occurred with the mini-implant position variable, where vestibular placement showed an I2 value of 47.55% (Q = 13.35; p = 0.06). Palatine/lingual placement obtained a lower value, I2 = 34.62% (Q = 10.71; p = 0.15). Lastly, ridge placement obtained I2 = 13.16 (Q = 5.75; p = 0.33), indicating that vestibular placement showed moderate heterogeneity, while lingual and crestal placement showed low heterogeneity. Odds ratios and confidence intervals showed that none of these variables reached statistical significance (vestibular odds ratio 0.82; CI = 0.56 to 1.19; lingual odds ratio 1.10; 95% CI = 0.78 to 1.55; ridge odds ratio 1.53; 95% CI = 0.95 to 2.45).

With regard to placement on the left side or right side, or in anterior or posterior regions, statistically significant relations were found for both variables (left/right side: odds ratio 1.128, with a 95% CI = 0.783 to 0.627; anterior/posterior location: odds ratio 1.48, with a 95% CI = 0.59 to 3.72). The I2 value showed an absence of heterogeneity; I2 values were 50.9 (Q = 16.30; p = 0.03), and I2 = 53.94 (Q = 8.68; p = 0.06), respectively.

Lastly, mini-implant dimension variables, length and diameter, did not obtain statistically significant relations (diameter: odds ratio 0.74, with a 95% CI = 0.44 to 1.25; length: odds ratio 0.78, with a 95% CI = 0.36 to 1.69). In both cases the I2 value indicated heterogeneity: I2 = 61.11 (Q = 10.28; p = 0.03) and I2 = 69.6 (Q = 9.87; p = 0.02).

### Publication bias

**Figure 2:**
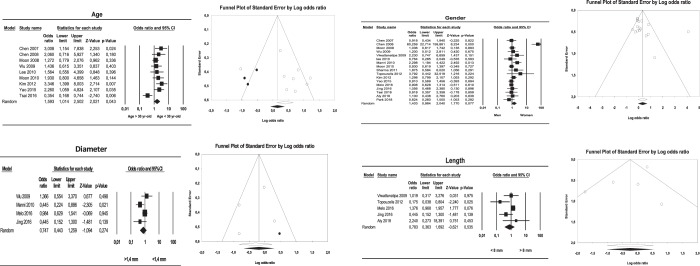
Forest and funnel plots for meta-analyses for: gender, age, mini-implant length and mini-implant diameter.

**Figure 3:**
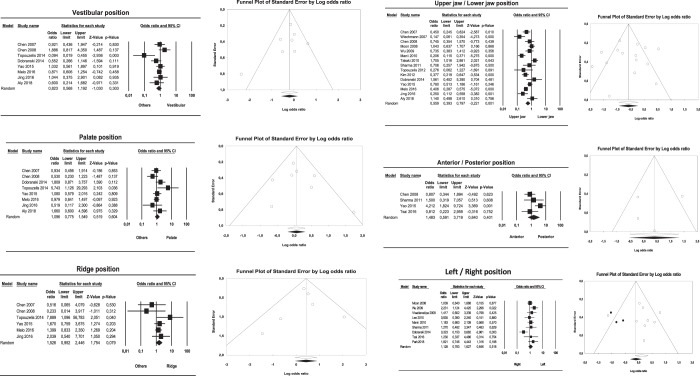
Forest and funnel plots for meta-analyses for different mini-implant locations.

Funnel plots presented symmetrical images without differences in estimations when imputed values were added, with the exception of crestal placement. The plot for this variable did show some asymmetry, indicating the existence of publication bias (Figs. 2 and 3).

None of the Egger’s regression intercepts calculated for the different Odds ratio estimations, have shown significance. For upper and lower jaw, the results were −0.37 between −3.87 and 3.21, p = 0.84; for gender 2.06 between −0.73 and 4.86, p = 0.13; for age 2.26 between −3.05 and 7.59, p = 0.34, vestibular position −1.1, p = 0.36; palatine/lingual 0.82 between −2.09 and 3.75, p = 0.51; ridge position −0.14 between −2.69 and 2.40, p = 0.88; age 2.26, p = 0.34; diameter −1.38, p = 0.631; length −1.45 between −4.95 and 2.04, p = 0.27; anterior and posterior position −1.10 between −19.1 and 15.3, p = 0.70; and right and left location 0.35, p = 0.85.

## Discussion

### Summary of evidence

The null hypothesis of the present study was rejected. When analyzing failure rates, significant differences were found when comparing different mini-implant locations. The maxillary location showed significantly lower failure rates than the mandibular, while the rest of the analyzed variables did not show significant effects.

Bone anchorage in orthodontic treatments is becoming ever more widespread. As the literature states repeatedly, the advantages offered by mini-implants are numerous, and the technique shows a success rate of over 80%^37^. But when mini-implants fail they do so during the first 8 weeks after placement, the period when implant stability changes from mechanical to biological stability^6^. The reasons for failure (mobility, displacement or infection of the surrounding soft tissues) were not assessed in the present literature review, as its objective was to determine which variables predispose mini-implants to failure.

Longitudinal studies, cohort and case/control studies both prospective and retrospective were included in the present review. Although RCTs were eligible to be included, none met the inclusion criteria. Cohort, case/control and RCT studies are the ones that better evaluate the risk factors associated with mini-implants stability. Including different study designs could lead to broad variations in outcome and quality terms. However, when analysing the quality of all of them we found that most of them present values between 6 and 7 in the *Newcastle-Ottawa scale* indicating that they have moderate quality **(**Table 3**)**.

Since weaker studies can influence the outcome of the present study, the sensitivity of the estimation of the 19 conducted meta-analyzes was analyzed and none of the studies was found to significantly influence the results, except one (Figs. 2 and 3).

The variables analyzed can be divided into three groups: variables deriving from the patient, others related to the mini-implant used (length and diameter), and a third set related to location and technique. Among the variables deriving from the patient, the present study identified some controversy surrounding the possible association between mini-implant failure and age. Some studies found no association between age and failure^14,15,32^, while others found that the patient’s age could influence mini-implant failure because younger patients present finer cortical bone and lower bone density^19,30–34^. (Fig. 2)

The variable sex was also subject to controversy. While some studies^12–14,30,31^ claim that the patients’ gender does not influence the success or failure of treatment with mini-implants, other works^33,35^ found a higher mini-implant success rate among men, which they attributed due to their higher bone density. However, the present review did not generally find a significant association between sex and loss of secondary stability.

Several other factors may influence failure/success rates but the present review was unable to analyze them due to the lack of published articles. These include poor hygiene, inflammation of surround tissues and the individual patient’s bone density^36^, all thought to play a role in treatment failure^13^.

When mini-implant characteristics were analyzed, those articles that analyzed different implant sizes^16,27,28^, agreed that diameters smaller than 1 mm show a tendency to failure. These findings make sense since smaller diameters provide less attachment surface and also, they are weaker so prone to fracture. But other studies^14,31^ found that length and diameter were not variables with any significant influence on success/failure rates. Meanwhile, two studies^11,24^ found that length was a factor that influences stability. The present meta-analysis did not show that mini-implants longer than 8 mm or with diameters over 1.4 mm undergo fewer failures than those with smaller dimensions.

Regarding mini-implant location, two studies^14,16^ stated that mini-implants presented a worse prognosis in posterior regions and in the alveolar mucosa. Between vestibular, lingual/palatine and crestal placement, two studies^16,26^ agreed that there is a higher risk of loss of mini-implant stability in lingual/palatine areas. But another study^24^ found that placement in crestal areas shows greater risk of failure. Meanwhile, two studies^31,34^ affirmed that the right side presents a higher risk of failure than the left. This could be due to patient’s oral hygiene maintenance capability or the dexterity of the clinician. The present review did not find significant relations between mini-implant failure and vestibular, lingual, or crestal placement or between placement on the left or right side. Only two articles^14,26^ differentiate jaw and maxilla together with the anterior and posterior position obtaining higher success rates in the anterior maxillary area (including the palate), with no statistically significant differences.

However, in agreement with some studies^33,35^ placement of mini-implants in the mandible could be a protective factor due to the mandible’s higher bone density. (Fig. 3)

Among the technical characteristics of mini-implant placement, there is also some controversy surrounding the intensity and duration of the force applied. One study^16^ claimed that the correct force is between 100 and 200 grams, and concluded that lower forces produced higher success rates, as did applying force for the shortest possible time. As for surgical technique, one work^28^ concluded that mini-implant placement without flap raising produces less damage and so higher success rates. Mini-implant placement using a motor also favored a successful outcome, moreover the surgical protocol with or without predrilling has only been assessed in a study that showed no significant differences. One study^33^ obtained a success rate of 88.38% for no-predrilling mini-implant insertion and 89.09% for predrilling, with an odds ratio of 0.93 (95% CI 0.36 to 2.42). For some authors, surgical technique is one of the most influential variables affecting stability^18^.

Knowledge of the factors involved in the failure of mini-implants can help the clinicians to improve their clinical practice. Within the analyzed factors there are some that could be adapted for a higher success. Only location seems to have a significant effect, having the upper jaw lower failure rate than the lower. However, this cannot be clinically modified since it depends on the biomechanics required in each case. The other analyzed factors did not show differences and their failure rates are very similar. (Table 4)

### Limitations

The limited number of studies that have investigated the variables analyzed in the present review could lead to estimation bias or even failure to identify their significance. Further prospective studies under controlled conditions could produce better information and improve the outcomes of orthodontic treatment involving the use of mini-implants.

## Conclusions

Based on the results of the present study, some variables both mini-implant and patient-dependent are related to the success rates of mini-implants (age, location and mini-implant length). The only significant factor was location, being upper maxilla placement of mini-implants more successful than mandible. The conclusions drawn from the present analysis regarding correlations between success/failure and the analyzed factors should be treated with caution due to the different methodologies employed in the different studies reviewed.
